# Response to induction chemotherapy predicts survival outcomes in oropharyngeal cancer

**DOI:** 10.1002/cam4.5656

**Published:** 2023-01-27

**Authors:** Qixian Zhang, Tingting Xu, Chunying Shen, Wei Qian, Hongmei Ying, Xiayun He, Yu Wang, Qinghai Ji, Chaosu Hu, Xin Zhou, Xueguan Lu

**Affiliations:** ^1^ Department of Radiation Oncology Fudan University Shanghai Cancer Center Shanghai China; ^2^ Department of Oncology Shanghai Medical College of Fudan University Shanghai China; ^3^ Shanghai Clinical Research Center for Radiation Oncology Shanghai China; ^4^ Shanghai Key Laboratory of Radiation Oncology Shanghai China; ^5^ Department of Head and Neck Surgery Fudan University Shanghai Cancer Center Shanghai China

**Keywords:** human papillomavirus, induction chemotherapy, oropharyngeal cancer, prognosis, treatment response

## Abstract

**Background:**

The role of induction chemotherapy (IC) in oropharyngeal squamous cell carcinoma (OPSCC) remains controversial. Its interpretation can be confounded by heterogeneity in chemosensitivity and human papillomavirus (HPV) status. This study aimed to investigate the prognostic impact of IC response in HPV‐positive and ‐negative OPSCC.

**Methods:**

Patients with OPSCC who underwent IC and concurrent chemoradiotherapy (CCRT) were retrospectively analyzed. Radiologic response to IC by ≥50% was defined as IC‐sensitive (IC‐s), while lesser response was deemed as IC‐resistant (IC‐r). Progression‐free survival (PFS) and overall survival (OS) were compared between subgroups.

**Results:**

A total of 51 HPV‐positive and 57 HPV‐negative patients were included. IC‐s patients accounted for 55.6%, 62.7%, and 49.1% in the entire cohort, HPV‐positive, and HPV‐negative subgroup, respectively. Compared with IC‐r subgroup, IC‐s was associated with better clinical outcomes either in the entire cohort (3y‐PFS 91.7%vs.43.7%, *P* < 0.001; 3y‐OS 98.3% vs. 67.4%, *P* = 0.002), the HPV‐positive subgroup (3‐year PFS 94.7% vs. 47.9%, *P* < 0.001; 3‐year OS 100% vs. 73.5%, P = 0.055) or the HPV‐negative subgroup (3‐year PFS 88.2% vs. 40.9%, *P* = 0.001; 3‐year OS 96.4% vs. 63.1%, *P* = 0.026). Multivariate analysis demonstrated that response to IC represents an independent prognosticator for 3‐year PFS (HR, 0.088; 95% CI, 0.027–0.289; *P* < 0.001) and 3‐year OS (HR, 0.100; 95% CI, 0.021–0.477; *P* = 0.004).

**Conclusions:**

Response to IC exerts a critical predictive effect on prognosis of both HPV‐positive and ‐negative OPSCC. Personalized treatment strategy based on IC response is worthy of further exploration in the future.

## Graphical abstract

Response to induction chemotherapy is an important predictor of prognosis in HPV‐positive and ‐negative oropharyngeal squamous cell carcinoma.

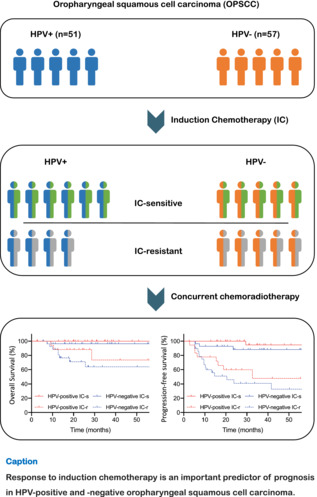

## 1 INTRODUCTION

According to the American Cancer Society estimation in 2022, there were 54,000 new patients with head and neck malignancies in the United States, accounting for 2.82% of all newly diagnosed cancer patients.^1^ As a distinct clinical subset of head and neck squamous cell carcinoma (HNSCC), oropharyngeal squamous cell carcinoma (OPSCC) can be divided into human papillomavirus (HPV)‐positive subtype caused by HPV infection and HPV‐negative subtype largely attributed to tobacco and alcohol consumption. The incidence of HPV‐positive OPSCC has significantly increased during the past decades.^2^ Compared with other HNSCC, HPV‐positive OPSCC has unique biological characteristics with a higher response rate for chemoradiotherapy and a more favorable prognosis than HPV‐negative counterparts,^3^, ^4^ which led to a separate classification with general downstage in the 8th edition of the American Joint Committee on Cancer (AJCC) staging system. Nevertheless, the clinical treatment strategies between HPV‐positive and HPV‐negative OPSCC remain indiscriminate now, where concurrent chemoradiotherapy (CCRT) stays as a major recommendation for locoregionally‐advanced disease.^5^ Whether there is room for personalized treatment still awaits further evidence.

Induction chemotherapy (IC) has been widely practiced, albeit with controversy, as an alternative option in locoregionally‐advanced HNSCC. For nonoropharyngeal HNSCC such as larynx and hypopharynx cancer, IC is well‐recognized with benefits in larynx preservation,^6^, ^7^, ^8^ although it seemed to yield no overall survival (OS) improvement in most randomized trials.^1^, ^9^, ^10^ In OPSCC, however, there is yet no consensus on the significance of IC due to the lack of solid evidence despite a category 3 recommendation in the National Comprehensive Cancer Network guidelines.^5^ Previous data failed to support the routine use of IC prior to CCRT by showing insignificant and even worse OS either in HPV‐positive or HPV‐negative OPSCC.^11^, ^12^ This perplexity and controversy highlight the potential heterogeneity of treatment effects among patients that might counteract the eventual benefit of IC, and careful selection of the target population most likely to profit from IC is extremely important to elucidate its real impact on long‐term outcomes.

The response to IC reflects the intrinsic chemosensitivity in vivo. Given the high concordance of chemosensitivity and radiosensitivity, the response to upfront chemotherapy has been widely used as a biomarker to select patients for subsequent adaptive radiotherapy in various malignancies including HNSCC.^13^, ^14^, ^15^ More importantly, emerging evidence revealed that a good response to IC can translate into a survival benefit. Subanalyzed results from two phase III prospective clinical trials demonstrated that the patients with oral cavity cancer with clinical response to IC had better prognoses than nonresponders.^16^, ^17^ A similar observation was found in paranasal sinuses squamous cell carcinoma, where the response to IC conferred improved oncologic outcome and organ preservation.^18^ However, the prognostic value of response to IC remains unknown in OPSCC, especially regarding HPV status. In this study, we aimed to explore the association of response to IC before CCRT with clinical outcomes in both HPV‐positive and HPV‐negative OPSCC at a single institution.

## 2 MATERIALS AND METHODS

### 2.1 Eligibility criteria and patient population

**FIGURE 1:**
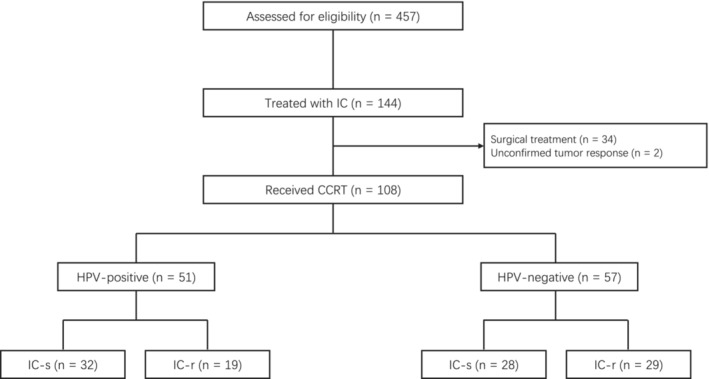
Patient flow diagram. CCRT, concurrent chemoradiotherapy; HPV, human papillomavirus; IC‐r, resistant to induction chemotherapy; IC‐s, sensitive to induction chemotherapy.

Patients with newly diagnosed OPSCC at Fudan University Shanghai Cancer Center between 2010 and 2020 were retrospectively reviewed under the approval of the institutional review board. Inclusion criteria were as follows: (1) treatment‐naïve and histologically confirmed OPSCC; (2) staged as II‐IVb; (3) HPV status available; (4) Karnofsky Performance Status Scale ≥70%; (5) received IC followed by concurrent chemoradiotherapy and (6) accessible pre‐ and post‐IC radiologic images for tumor evaluation (the workflow of patient selection is shown in Figure 1). HPV status was confirmed through p16 immunohistochemistry and p16 expression in ≥70% of tumor cells was recognized as HPV‐positive. All staging was conducted according to the 7th edition of the International Union against Cancer/American Joint Committee on Cancer (UICC/AJCC) staging system, based on a baseline workup including thorough physical examination, laboratory tests, and radiologic imaging for evaluation of loco‐regional diseases and exclusion of distant metastases.

### 2.2 Treatment and tumor assessment

Two or three cycles of IC were intended with TPF (docetaxel 75 mg/m^2^ on d1, cisplatin 75–80 mg/m^2^ on day 1 and 5‐fluorouracil 500 mg/m^2^ with continuous infusion on days 1–5), TP (docetaxel 75 mg/m^2^ on day 1 and cisplatin 75–80 mg/m^2^ on day 1) or PF (cisplatin 75–80 mg/m^2^ on day 1 and 5‐fluorouracil on days 1–5) regimen. CCRT started within 4 weeks after IC, consisting of intensity‐modulated radiotherapy (IMRT) with a radical dose of 60–70Gy in 30–35 fractions to the gross tumor, and cisplatin of 75–80 mg/m^2^ concurrently given every 3 weeks.

Tumor response to IC was evaluated according to the Response Evaluation Criteria in Solid Tumors version 1.1^19^ on cross‐sectional MR or CT images at 2–3 weeks after the completion of IC. Major PR referred to a ≥ 50% decrease in both primary and lymph node sites. Minor PR represented a 30%–50% decrease. By further dividing partial response (PR) into major PR (response by ≥50%) and minor PR (response by 30%–50%), we defined an IC‐sensitive subgroup (IC‐s) with complete response (CR) or major partial response (PR), and an IC‐resistant subgroup (IC‐r) with suboptimal response including minor PR, stable disease (SD), or progressive disease (PD).

### 2.3 Endpoints and statistics

Endpoints were calculated from the initiation of the first IC, including progression‐free survival (PFS, time to tumor relapse or death from any cause), overall survival (OS, time to death from any cause), local recurrence‐free survival (LFS, time to local relapse or death from any cause), regional recurrence‐free survival (RFS, time to regional recurrence or death from any cause), and distant metastasis‐free survival (DMFS, time to distant metastasis or death from any cause).

Statistical analysis was performed using the SPSS software (version 26.0; SPSS, Chicago, IL). Categorical variables were compared between groups with the chi‐squared test or Fisher's exact test. Kaplan–Meier method followed by the log‐rank test was used to estimate and compare the survival outcomes. Cox proportional hazards model was used for the multivariate survival analysis. A value of *P* < 0.05 was considered to be statistically significant.

## 3 RESULTS

### 3.1 Patient cohort

In total, 457 patients were assessed for eligibility, of which 144 received IC. After excluding 34 patients who underwent surgery and two patients whose tumor response status could not be determined, 108 OPSCC patients met the inclusion criteria including 51 with HPV‐positive and 57 with HPV‐negative status. The baseline characteristics of the patients are shown in Table 1. The median age was 58 years (range, 23 to 76 years). The male‐to‐female ratio was 4.35 to 1. One hundred and five patients (97.2%) had stage III‐IV disease, with the most common primary site at the tonsil (59.2%). TP regimen was administered in 88.9% of patients. Compared with HPV‐positive OPSCC, the HPV‐negative group tended to be younger (median age, 58 vs. 54 years), have more males (51/57 vs. 37/51, *P* = 0.022), heavier smokers (≥ 10 packs/year, 35/57 vs. 14/51, *P* < 0.001) and more nontonsil primary origins (33/57 vs. 11/51, *P* < 0.001). The IC‐s subgroup of the HPV‐negative patients had a higher percentage of low N stage (N0‐N1, 9/28 vs. 2/29, *P* = 0.038) than the corresponding IC‐r subgroup.

**TABLE 1:** Patient characteristics.

	All patients (*n* = 108)			HPV‐positive patients (*n* = 51)			HPV‐negative patients (*n* = 57)		
Characteristic	IC‐s (*n* = 60)	IC‐r (*n* = 48)	*P*	IC‐s (*n* = 32)	IC‐r (*n* = 19)	*P*	IC‐s (*n* = 28)	IC‐r (*n* = 29)	*P*
Follow‐up time, median, months	29.6			30.9			29.6		
Age, *n* (%)									
Age < 58 years	30 (50.0)	19 (39.6)	0.280	17 (53.1)	10 (52.6)	0.973	13 (46.4)	9 (31.0)	0.233
Age ≥ 58 years	30 (50.0)	29 (60.4)		15 (46.9)	9 (47.4)		15 (53.6)	20 (69.0)	
Gender, *n* (%)									
Male	47 (78.3)	40 (83.3)	0.514	22 (68.8)	14 (73.7)	0.708	25 (89.3)	26 (89.7)	0.964
Female	13 (21.7)	8 (16.7)		10 (31.3)	5 (26.3)		3 (10.7)	3 (10.3)	
Smoking, *n* (%)									
≥ 10 packs/year	29 (48.3)	20 (41.7)	0.775	10 (31.3)	4 (21.1)	0.726	19 (67.9)	16 (55.2)	0.615
< 10 packs/year	5 (8.3)	5 (10.4)		3 (9.4)	2 (10.5)		2 (7.1)	3 (10.3)	
Never/unknown	26 (43.3)	23 (47.9)		19 (59.4)	13 (68.4)		7 (25.0)	10 (34.5)	
Primary site, *n* (%)									
Palatine tonsil	34 (56.7)	30 (62.5)	0.909	23 (71.9)	17 (89.5)	0.267	11 (39.3)	13 (44.8)	0.967
Base of tongue	17 (28.3)	11 (22.9)		7 (21.9)	1 (5.3)		10 (35.7)	10 (34.5)	
Soft palate	2 (3.3)	2 (4.2)		0 (0.0)	0 (0.0)		2 (7.1)	2 (6.9)	
Pharyngeal wall	7 (11.7)	5 (10.4)		2 (6.3)	1 (5.3)		5 (17.9)	4 (13.8)	
T stage, *n* (%)									
T1‐T2	37 (61.7)	28 (58.3)	0.725	20 (62.5)	11 (57.9)	0.745	17 (60.7)	17 (58.6)	0.872
T3‐T4	23 (38.3)	20 (41.7)		12 (37.5)	8 (42.1)		11 (39.3)	12 (41.4)	
N stage, *n* (%)									
N0‐N1	15 (25.0)	9 (18.8)	0.438	6 (18.8)	7 (63.2)	0.271	9 (32.1)	2 (6.9)	0.038
N2‐N3	45 (75.0)	39 (81.3)		26 (81.3)	12 (36.8)		19 (67.9)	27 (93.1)	
IC, *n* (%)									
≤2	45 (75.0)	30 (62.5)	0.161	22 (68.8)	13 (68.4)	0.980	23 (82.1)	17 (58.6)	0.052
>2	15 (25.0)	19 (37.5)		10 (31.3)	6 (31.6)		5 (17.9)	12 (41.4)	
IC regimen, *n* (%)									
TP	54 (90.0)	42 (87.5)	0.734	29 (90.6)	16 (84.2)	0.595	25 (89.3)	26 (89.7)	0.357
TPF	1 (1.7)	2 (4.2)		1 (3.1)	0 (0.0)		0 (0.0)	2 (6.9)	
Other regimens	5 (8.3)	4 (8.3)		2 (6.3)	3 (15.8)		3 (10.7)	1 (3.4)	
Radiation dose, Gy									
Median	70	70		70	70		70	70	
Range	60 to 70.4	66 to 70.4		60 to 70.4	66 to 70.4		60 to 70	66 to 70	

Abbreviations: HPV, human papillomavirus; IC‐r, resistant to induction chemotherapy; IC‐s, sensitive to induction chemotherapy.

### 3.2 Tumor responses to IC

For the primary tumor, 45 of 108 patients (41.7%) achieved a CR, 39 (36.1%) achieved a major PR, 14 (13.0%) achieved a minor PR, 9 (8.3%) had SD and 1 (0.9%) had PD. For regional nodes, a CR in 21 (19.4%) patients, major PR in 43 (39.8%), minor PR in 18 (16.7%), SD in 25 (23.1%) and PD in 1 (0.9%) was observed. In total, the response to IC included CR in 12 (11.1%), major PR in 48 (44.4%), minor PR in 20 (18.5%), SD in 27 (25.0%), and PD in 1 (0.9%) patients. According to our definition, high sensitivity to IC was identified in 77.8% of patients for primary sites and 59.3% for lymph nodes, leading to an overall sensitivity of 55.6% (Table 2) in the entire cohort.

**TABLE 2:** Response to induction chemotherapy.

	All patients (*n* = 108)			HPV‐positive patients (*n* = 51)			HPV‐negative patients (*n* = 57)		
	OP	LN	OP+LN	OP	LN	OP+LN	OP	LN	OP+LN
IC‐s, n (%)	84 (77.8%)	64 (59.3%)	60 (55.6%)	44 (86.3%)	34 (66.7%)	32 (62.7%)	40 (70.2%)	30 (52.6%)	28 (49.1%)
CR	45 (41.7%)	21 (19.4%)	12 (11.1%)	26 (51.0%)	13 (25.5%)	7 (13.7%)	19 (33.3%)	8 (14.0%)	5 (8.8%)
Major PR	39 (36.1%)	43 (39.8%)	48 (44.4%)	18 (35.3%)	21 (41.2%)	25 (49.0%)	21 (36.8%)	22 (38.6%)	23 (40.4%)
IC‐r, n (%)	24 (22.2%)	44 (40.7%)	48 (44.4%)	7 (13.7%)	17 (33.3%)	19 (37.3%)	17 (29.8%)	27 (47.4%)	29 (50.9%)
Minor PR	14 (13.0%)	18 (16.7%)	20 (18.5%)	2 (3.9%)	5 (9.8%)	5 (9.8%)	12 (21.1%)	13 (22.8%)	15 (26.3%)
SD	9 (8.3%)	25 (23.1%)	27 (25.0%)	5 (9.8%)	12 (23.5%)	14 (27.5%)	4 (7.0%)	13 (22.8%)	13 (22.8%)
PD	1 (0.9%)	1 (0.9%)	1 (0.9%)	0 (0.0%)	0 (0.0%)	0 (0.0%)	1 (1.8%)	1 (1.8%)	1 (1.8%)

Abbreviation: CR, complete response; IC‐r, resistant to induction chemotherapy; IC‐s, sensitive to induction chemotherapy; LN, lymph node; OP, oropharynx; ORR, objective response rate; PD, progressive disease; PR, partial response; SD, stable disease.

The overall distribution of response between HPV‐positive and HPV‐negative patients was insignificant. However, the HPV‐positive subgroup did show a higher rate of CR (51.0% vs. 33.3% in oropharynx, 25.5% vs. 14.0% in nodes and 13.7% vs. 8.8% in total) and IC‐s (86.3% vs. 70.2% in oropharynx, 66.7% vs. 52.6% in nodes and 62.7% vs. 49.1% in total) when compared with HPV‐negative patients, suggesting that the HPV‐positive OPSCC is more sensitive to upfront chemotherapy. No differences in IC sensitivity were found in terms of age, gender, smoking history, primary site, or IC regimen.

### 3.3 Survival outcomes

The median follow‐up was 29.6 months (range, 2.6 to 153.7 months). Overall, 29 patients (26.9%) in the entire cohort developed tumor relapse, including 13.0% local, 11.1% regional recurrence, and 8.3% distant metastasis. The 3‐year OS and PFS were 90.0% and 71.4%, respectively.

**FIGURE 2:**
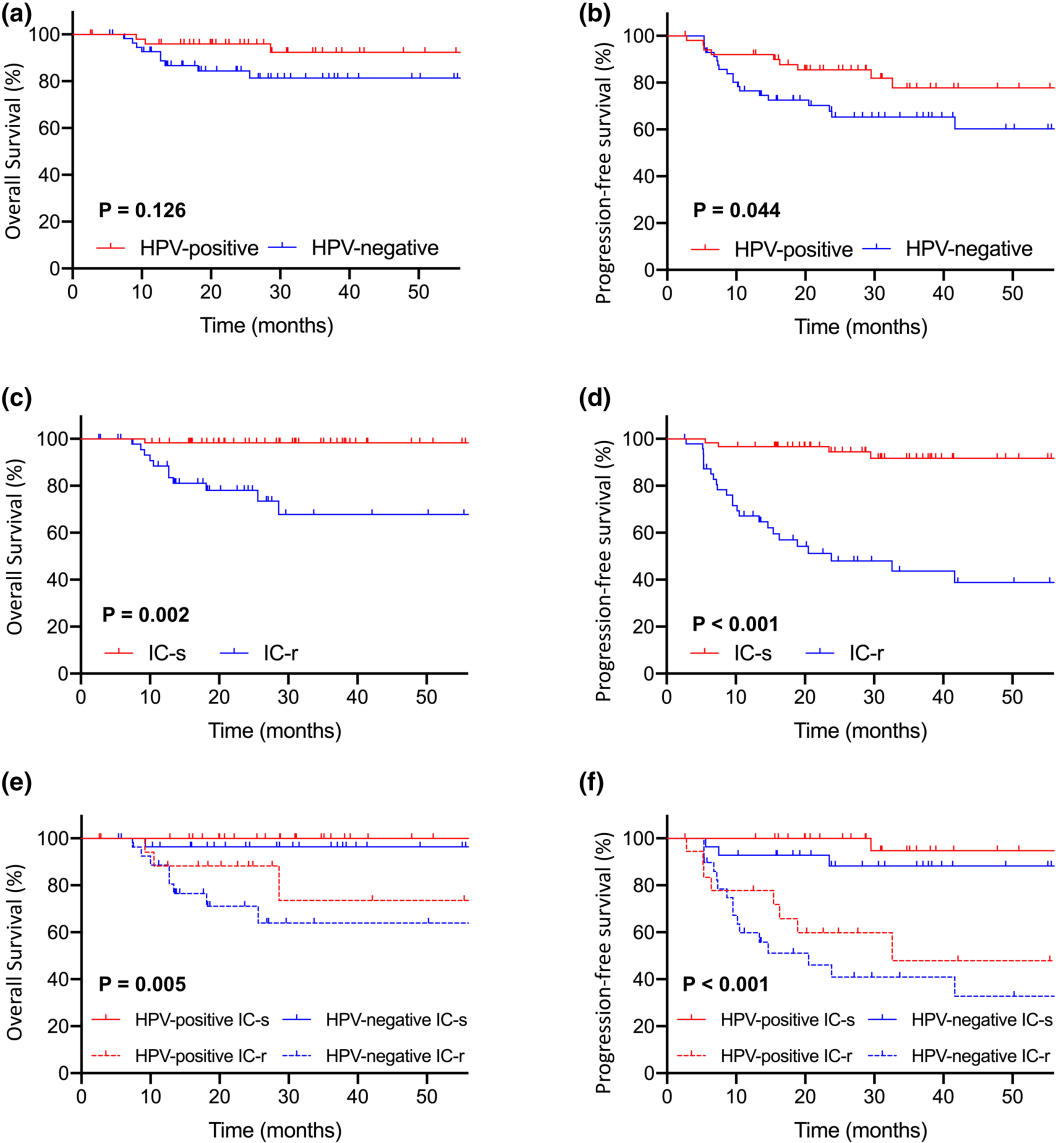
(A) OS based on HPV status in the entire cohort. (B) PFS based on HPV status in the entire cohort. (C) OS based on response to IC in the entire cohort. (D) PFS based on response to IC in the entire cohort. (E) OS based on HPV status and response to IC. (F) PFS based on HPV status and response to IC. HPV, human papillomavirus; IC‐r, resistant to induction chemotherapy; IC‐s, sensitive to induction chemotherapy; PFS, progression‐free survival; OS, overall survival.

The 3‐year OS and PFS for HPV‐positive OPSCC were 92.4% and 77.8%, respectively, compared with 81.4% and 65.3% for the HPV‐negative subgroup, respectively. HPV‐positive OPSCC was found to have improved PFS but not OS than the HPV‐negative OPSCC (Figure 2A,B).

For the entire population, there was no obvious survival difference between heavy smokers and nonheavy smokers (3‐year OS 83.8% vs. 89.0%, *P* = 0.390). Patients with low T stage (T1‐T2) showed prolonged OS and PFS (3‐year OS 95.0% vs. 74.4%, *P* = 0.001; 3‐year PFS 78.0% vs. 62.1%, *P* = 0.044) compared with the high T stage (T3‐T4) cohort.

### 3.4 Prognostic impact of response to IC

Compared with the IC‐r patients, the IC‐s subgroup had more favorable 3‐year OS (98.3% vs. 67.4%, *P* = 0.002, Figure 2C), PFS (91.7% vs. 43.7%, *P* < 0.001, Figure 2D), LFS (94.4% vs. 74.1%, *P* = 0.008), RFS (100.0% vs. 68.6%, *P* < 0.001), and DMFS (94.9% vs. 78.6%, *P* = 0.006) (Figure S1). In HPV‐positive OPSCC, IC‐s was associated with a significantly higher 3‐year PFS (94.7% vs. 47.9%, *P* < 0.001) and a marginally improved OS (100% vs. 73.5%, *P* = 0.055). The major survival benefit lay in the loco‐regional tumor control (3‐year LFS 100.0% vs. 80.9%, *P* = 0.013; 3‐year RFS 100.0% vs. 70.2%, *P* = 0.009). Similar effects with PFS and OS were observed in the HPV‐negative subgroup (3‐year PFS 88.2% vs. 40.9%, *P* = 0.001; 3‐year OS 96.4% vs. 63.1%, *P* = 0.026), whereas the improvement was seen mainly in regional and distant control (3‐year RFS 100.0% vs. 66.9%, *P* = 0.007; 3‐year DMFS 95.0% vs. 72.1%, *P* = 0.029) (Figure S1). Interestingly, HPV status seemed to lose its effect in separating survivals when regarding IC response, HPV‐positive and HPV‐negative patients showed no significant difference in 3‐year OS and PFS either in IC‐s or IC‐r subgroups (Figure 2E,F).

In the entire cohort, factors associated with OS in the univariate analysis included T stage and response to IC, while gender, HPV status, T stage, and response to IC were associated with PFS (Table 3). On multivariable Cox regression, IC‐s remained an independent prognosticator for superior PFS (hazard ratio [HR], 0.088; 95% confidence interval [CI], 0.027–0.289; *P* < 0.001) and OS (HR, 0.100; 95% CI, 0.021–0.477; *P* = 0.004). In subset analyses, the multivariable Cox regression showed that IC‐s was significantly associated with PFS (HR, 0.079; 95% CI, 0.020 to 0.315; *P* < 0.001) and OS (HR, 0.050; 95% CI, 0.005 to 0.510; *P* = 0.012) in HPV‐negative OPSCC, as well as PFS (HR, 0.053; 95% CI, 0.006 to 0.450; *P* = 0.007) in HPV‐positive OPSCC. However, the response to IC failed to predict OS in HPV‐positive OPSCC (Table 3).

**TABLE 3:** Three‐year overall survival and progression‐free survival: univariate and multivariate Cox Proportional Hazards Models.

	Univariate		Multivariate	
Variable	HR (95% CI)	*P*	HR (95% CI)	*P*
Entire study population				
Overall survival				
Age (<58 vs. ≥ 58 y)	0.450 (0.140 to 1.441)	0.159		
Gender (male vs. female)	0.303 (0.039 to 2.328)	0.174		
Smoking (< 10 vs. ≥10 PKY)	0.631 (0.219 to 1.821)	0.391		
HPV (positive vs. negative)	0.415 (0.130 to 1.327)	0.119	0.321 (0.081 to 1.274)	0.106
IC response (IC‐s vs. IC‐r)	0.100 (0.021 to 0.477)	**0.004**	0.100 (0.021 to 0.477)	**0.004**
T stage (T1‐T2 vs. T3‐T4)	0.155 (0.043 to 0.560)	**0.001**	0.121 (0.027 to 0.532)	**0.005**
N stage (N0‐N1 vs. N2‐N3)	1.151 (0.318 to 4.163)	0.832	3.141 (0.602 to 16.401)	0.175
Progression‐free survival				
Age (<58 vs. ≥ 58 y)	0.812 (0.388 to 1.702)	0.580		
Gender (male vs. female)	0.260 (0.062 to 1.096)	**0.026**	0.182 (0.036 to 0.913)	**0.038**
Smoking (< 10 vs. ≥10 PKY)	0.701 (0.325 to 1.509)	0.356		
HPV (positive vs. negative)	0.454 (0.207 to 0.999)	**0.041**	0.466 (0.195 to 1.117)	0.087
IC response (IC‐s vs. IC‐r)	0.188 (0.027 to 0.289)	**< 0.001**	0.088 (0.027 to 0.289)	**< 0.001**
T stage (T1‐T2 vs. T3‐T4)	0.479 (0.230 to 0.998)	**0.049**	0.444 (0.200 to 0.983)	**0.045**
N stage (N0‐N1 vs. N2‐N3)	1.183 (0.504 to 2.777)	0.703	2.562 (0.946 to 6.939)	0.064
HPV+ patients				
Overall survival				
Age (<58 vs. ≥ 58 y)	0.986 (0.137 to 7.086)	0.988		
Gender (male vs. female)	0.920 (0.095 to 8.941)	0.942		
Smoking (< 10 vs. ≥10 PKY)	1.355 (0.141 to 13.058)	0.788		
IC response (IC‐s vs. IC‐r)	0.145 (0.015 to 1.414)	0.068	0.161 (0.014 to 1.814)	0.139
T stage (T1‐T2 vs. T3‐T4)	0.208 (0.022 to 2.011)	0.138	0.178 (0.012 to 2.573)	0.205
N stage (N0‐N1 vs. N2‐N3)	1.283 (0.130 to 12.667)	0.834	0.872 (0.044 to 17.246)	0.928
Progression‐free survival				
Age (<58 vs. ≥ 58 y)	2.025 (0.503 to 8.149)	0.306		
Gender (male vs. female)	0.301 (0.038 to 2.413)	0.189		
Smoking (< 10 vs. ≥10 PKY)	1.365 (0.283 to 6.575)	0.690		
IC response (IC‐s vs. IC‐r)	0.052 (0.006 to 0.415)	**<0.001**	0.053 (0.006 to 0.450)	**0.007**
T stage (T1‐T2 vs. T3‐T4)	0.521 (0.140 to 1.944)	0.330	0.480 (0.116 yo 1.991)	0.312
N stage (N0‐N1 vs. N2‐N3)	1.577 (0.393 to 6.323)	0.532	1.936 (0.407 to 9.205)	0.406
HPV‐ patients				
Overall survival				
Age (<58 vs. ≥ 58 y)	0.367 (0.078 to 1.733)	0.167		
Gender (male vs. female)	0.039 (0.000 to 164.275)	0.112		
Smoking (< 10 vs. ≥10 PKY)	0.619 (0.158 to 2.430)	0.480		
IC response (IC‐s vs. IC‐r)	0.203 (0.043 to 0.964)	**0.023**	0.050 (0.005 to 0.510)	**0.012**
T stage (T1‐T2 vs. T3‐T4)	0.134 (0.028 to 0.641)	**0.004**	0.126 (0.018 to 0.892)	**0.038**
N stage (N0‐N1 vs. N2‐N3)	1.205 (0.250 to 5.813)	0.819	9.311 (0.759 to 144.291)	0.081
Progression‐free survival				
Age (<58 vs. ≥ 58 y)	0.582 (0.223 to 1.521)	0.255		
Gender (male vs. female)	0.299 (0.040 to 2.252)	0.159		
Smoking (< 10 vs. ≥10 PKY)	0.907 (0.346 to 2.378)	0.842		
IC response (IC‐s vs. IC‐r)	0.177 (0.059 to 0.534)	**<0.001**	0.079 (0.020 to 0.315)	**<0.001**
T stage (T1‐T2 vs. T3‐T4)	0.447 (0.183 to 1.088)	0.076	0.435 (0.155 to 1.218)	0.113
N stage (N0‐N1 vs. N2‐N3)	1.084 (0.358 to 3.277)	0.888	4.030 (0.922 to 17.611)	0.064

*Note*: Bold values in indicate that the corresponding variables (e.g. IC‐s, T1‐T2) are associated with improved OS or PFS.

Abbreviations: CI, confidence interval; HPV, human papillomavirus; HR, hazard ratio; IC‐r, resistant to induction chemotherapy; IC‐s, sensitive to induction chemotherapy; OS, overall survival; PFS, progression‐free survival. PKY, packs/year.

## 4 DISCUSSION

Since the first report from RTOG 0129, HPV status has been recognized as an independent prognostic indicator in OPSCC.^20^ According to Fakhry et al.^21^ HPV‐positive laryngeal and oropharyngeal cancers had a higher response rate to IC compared with HPV‐negative counterparts. However, evidence is lacking whether this is directly related to improved prognosis. Our data showed that HPV‐positive OPSCC experienced more CR and major PR after IC. Good response to IC led to improved OS and PFS in both HPV‐positive and HPV‐negative OPSCC. To our knowledge, this is the first report that thoroughly illustrated the interplay among HPV status, chemosensitivity and prognosis in OPSCC.

To achieve better organ preservation, IC followed by CCRT has been recommended as a nonsurgical alternative in locoregionally advanced HNSCC. In squamous cell carcinoma of paranasal sinuses, those who acquired objective response after platinum‐based IC had significantly better 2‐year disease‐free survival and OS.^18^ A phase III trial in oral cancer found no survival benefits from IC before surgery in the entire population, but a subgroup of patients with a favorable pathological response after TPF induction had notably higher DFS and OS when compared with the control arm and those with unfavorable response.^22^ In OPSCC, the effect of IC in the setting of CCRT is more perplexing. Sher et al.^11^ found no significant difference in OS between CCRT and IC plus CCRT. In another retrospective study, IC was predictive of even worse OS and DMFS in OPSCC, especially HPV‐positive OPSCC.^12^ By contrast, Bhattasali et al.^23^ reported that IC remarkably improved PFS and DMFS in a selective high‐risk subgroup of p16‐positive OPSCC with low‐neck and/or N3 disease. In the present study, HPV‐positive and HPV‐negative subsets showed distinct survival rates, suggesting significant heterogeneity in OPSCC independent of HPV status. Suboptimal IC response and worse outcomes in the HPV‐positive OPSCC subpopulation challenged the stereotypes that this entity always exhibits a good prognosis. We are attempting to clarify the genomic and transcriptomic predictors for IC sensitivity in an ongoing phase II trial (IChoice‐01, Clinical Trials. No: NCT04012502).

There were several interesting findings in the present study. First, the objective response rate (ORR) of HPV‐negative OPSCC was unexpectedly higher (75.4%) than that in the previous series (55%),^15^ which led to narrowed disparities in IC response between HPV‐positive and HPV‐negative OPSCC. Plausible explanations may include a relatively lower cigarette consumption and a higher rate of primary subsite in the tonsil and base of the tongue in our HPV‐negative patients, as well as potential genetic discrepancies between the Chinese and the Western population. Second, HPV‐negative OPSCC seemed to benefit more from IC. Although there was no direct comparison to CCRT in our study, the addition of IC in HPV‐negative OPSCC resulted in superior survivals to historical CCRT data from RTOG 0129^4^ and RTOG 0522^24^ (3‐year OS, 81.4% vs. 57.1% and 60.1%, respectively; 3‐year PFS, 65.3% vs. 43.4% and 49.2%, respectively). This was consistent with De Felice's report that favored the use of IC in HPV‐negative OPSCC with increased DMFS.^25^ Third, the prognostic impact of HPV status was overridden by the response to IC. Despite a trend towards favorable PFS and OS in HPV‐positive OPSCC, the difference between HPV‐positive and HPV‐negative subsets was insignificant in both IC‐s and IC‐r groups, indicating that IC response rather than HPV status might be more important for risk stratification. Based on our findings, future directions of personalized treatment may involve not only de‐intensification in good IC responders, but also intensification in poor responders with more effective agents such as immune checkpoint inhibitors to overcome the resistance to chemoradiation, either in HPV‐positive or HPV‐negative OPSCC.

Due to the retrospective nature of the present study, selection bias was inevitable and the results should be carefully interpreted. As the data were obtained from a single institution and the number of patients was limited, external validations with larger cohorts will be needed in the future. In addition, the effect of different IC regimens on tumor response and prognosis could not be analyzed because most patients received TP duplets. TPF has been established as the standard IC regimen due to more effective locoregional control over PF.^26^, ^27^ However, multiple studies have shown that this triplet regimen is accompanied by more acute toxicities that could compromise the tolerance for subsequent treatment.^28^, ^29^, ^30^TP regimen, in comparison, is a more patient‐friendly modality that comes with high tolerance and satisfactory efficacy in several studies. The trade‐off between chemo‐response and tolerance should be carefully weighed in clinical practice.

In the 8th edition of AJCC guidelines, p16 immunohistochemistry was recommended as a surrogate marker due to its high consistency with HPV status determined by E6/E7 mRNA expression (the “golden standard”) or HPV ISH.^31^, ^32^ It has been realized that p16 has higher sensitivity but relatively lower specificity when compared to HPV determined by ISH. The p16‐positive patients might include a subgroup with negative HPV by ISH, which was reported with an inferior prognosis with real HPV‐positive ones. By contrast, HPV ISH alone might underestimate 12%–15% of real HPV‐positive patients, leading to misjudged prognosis in these cases. Therefore, neither p16 nor HPV ISH alone is perfect diagnostic marker alone, and growing evidence supports their combination. As this retrospective study covered a wide time span from 2010 to 2020, HPV ISH data were not available for a small number of patients. Although p16 overexpression cannot predict the presence of HPV status adequately, it was chosen as the criterion for grouping considering the integrity and accessibility of p16 staining data.

In conclusion, this study represents the first attempt to demonstrate the prognostic impact of IC response in both HPV‐positive and HPV‐negative OPSCC. The response to IC provides a tool for better risk stratification and identification of candidates for subsequent treatment de‐intensification or intensification.

## AUTHOR CONTRIBUTIONS

Qixian Zhang, Tingting Xu, and Chunying Shen were involved in data curation, formal analysis, investigation, methodology, validation and writing—original draft. Wei Qian, Hongmei Ying, Xiayun He, and Yu Wang were involved in methodology, validation and writing—original draft. Qinghai Ji was involved in conceptualization, methodology, validation and writing—original draft. Chaosu Hu, Xin Zhou, and Xueguan Lu were involved in conceptualization, resources, funding acquisition, project administration, supervision and writing—review and editing.

## FUNDING INFORMATION

This study was supported by grants from Fudan University Shanghai Cancer Center Foundation Project (YJRC1903).

## CONFLICT OF INTEREST STATEMENT

The authors declare that there is no conflict of interests regarding the publication of this paper.

## ETHICS STATEMENT

This study was exempt from institutional review board approval due to the nature of the study. Because all data were de‐ identified, patient consent was waived.

## Supporting information


Figure S1.

## Data Availability

The data that support the findings of this study are available from the corresponding author upon reasonable request.

## References

[1] Gau M , Karabajakian A , Reverdy T , Neidhardt EM , Fayette J . Induction chemotherapy in head and neck cancers: results and controversies. Oral Oncol. 2019;95:164‐169. doi:10.1016/j.oraloncology.2019.06.015 31345386

[2] Chaturvedi AK , Engels EA , Pfeiffer RM , et al. Human papillomavirus and rising oropharyngeal cancer incidence in the United States. J Clin Oncol. 2011;29:4294‐4301. doi:10.1200/jco.2011.36.4596 21969503 PMC3221528

[3] Comprehensive genomic characterization of head and neck squamous cell carcinomas. Nature. 2015;517:576‐582. doi:10.1038/nature14129 25631445 PMC4311405

[4] Ang KK , Harris J , Wheeler R , et al. Human papillomavirus and survival of patients with oropharyngeal cancer. N Engl J Med. 2010;363:24‐35. doi:10.1056/NEJMoa0912217 20530316 PMC2943767

[5] Network NCC . (NCCN) clinical practice guidelines in oncology. Head and Neck Cancer, Version 1. (2022)

[6] Pointreau Y , Garaud P , Chapet S , et al. Randomized trial of induction chemotherapy with cisplatin and 5‐fluorouracil with or without docetaxel for larynx preservation. J Natl Cancer Inst. 2009;101:498‐506. doi:10.1093/jnci/djp007 19318632

[7] Lefebvre JL , Andry G , Chevalier D , et al. Laryngeal preservation with induction chemotherapy for hypopharyngeal squamous cell carcinoma: 10‐year results of EORTC trial 24891. Ann Oncol. 2012;23:2708‐2714. doi:10.1093/annonc/mds065 22492697 PMC3457747

[8] Forastiere AA , Zhang Q , Weber RS , et al. Long‐term results of RTOG 91‐11: a comparison of three nonsurgical treatment strategies to preserve the larynx in patients with locally advanced larynx cancer. J Clin Oncol. 2013;31:845‐852. doi:10.1200/jco.2012.43.6097 23182993 PMC3577950

[9] Petit C , Lacas B , Pignon JP , et al. Chemotherapy and radiotherapy in locally advanced head and neck cancer: an individual patient data network meta‐analysis. Lancet Oncol. 2021;22:727‐736. doi:10.1016/s1470-2045(21)00076-0 33862002

[10] Pignon JP , Bourhis J , Domenge C , Designé L . Chemotherapy added to locoregional treatment for head and neck squamous‐cell carcinoma: three meta‐analyses of updated individual data. MACH‐NC collaborative group. Meta‐analysis of chemotherapy on head and neck cancer. Lancet. 2000;355:949‐955. 10768432

[11] Sher DJ , Schwartz DL , Nedzi L , et al. Comparative effectiveness of induction chemotherapy for oropharyngeal squamous cell carcinoma: a population‐based analysis. Oral Oncol. 2016;54:58‐67. doi:10.1016/j.oraloncology.2015.12.008 26794877

[12] Guo TW , Saiyed F , Yao C , et al. Outcomes of patients with oropharyngeal squamous cell carcinoma treated with induction chemotherapy followed by concurrent chemoradiation compared with those treated with concurrent chemoradiation. Cancer. 2021;127:2916‐2925. doi:10.1002/cncr.33491 33873251 PMC11970557

[13] Lee DS , Lim DH , Kim IH , et al. Upfront chemotherapy followed by response adaptive radiotherapy for intracranial germinoma: prospective multicenter cohort study. Radiother Oncol. 2019;138:180‐186. doi:10.1016/j.radonc.2019.06.002 31319280

[14] Marur S , Li S , Cmelak AJ , et al. E1308: phase II trial of induction chemotherapy followed by reduced‐dose radiation and weekly cetuximab in patients with HPV‐associated Resectable squamous cell carcinoma of the oropharynx‐ ECOG‐ACRIN cancer research group. J Clin Oncol. 2017;35:490‐497. doi:10.1200/jco.2016.68.3300 28029303 PMC5455313

[15] Jouin A , Helfre S , Bolle S , et al. Adapted strategy to tumor response in childhood nasopharyngeal carcinoma: the French experience. Strahlenther Onkol. 2019;195:504‐516. doi:10.1007/s00066-019-01461-6 30963203

[16] Zhong LP , Zhang CP , Ren GX , et al. Randomized phase III trial of induction chemotherapy with docetaxel, cisplatin, and fluorouracil followed by surgery versus up‐front surgery in locally advanced resectable oral squamous cell carcinoma. J Clin Oncol. 2013;31:744‐751. doi:10.1200/jco.2012.43.8820 23129742 PMC5569675

[17] Bossi P , Lo Vullo S , Guzzo M , et al. Preoperative chemotherapy in advanced resectable OCSCC: long‐term results of a randomized phase III trial. Ann Oncol. 2014;25:462‐466. doi:10.1093/annonc/mdt555 24401930

[18] Abdelmeguid AS , Teeramatwanich W , Roberts DB , et al. Neoadjuvant chemotherapy for locoregionally advanced squamous cell carcinoma of the paranasal sinuses. Cancer. 2021;127:1788‐1795. doi:10.1002/cncr.33452 33567468

[19] Eisenhauer EA , Therasse P , Bogaerts J , et al. New response evaluation criteria in solid tumours: revised RECIST guideline (version 1.1). Eur J Cancer. 2009;45:228‐247. doi:10.1016/j.ejca.2008.10.026 19097774

[20] You EL , Henry M , Zeitouni AG . Human papillomavirus‐associated oropharyngeal cancer: review of current evidence and management. Curr Oncol. 2019;26:119‐123. doi:10.3747/co.26.4819 31043814 PMC6476447

[21] Fakhry C , Westra WH , Li S , et al. Improved survival of patients with human papillomavirus‐positive head and neck squamous cell carcinoma in a prospective clinical trial. J Natl Cancer Inst. 2008;100:261‐269. doi:10.1093/jnci/djn011 18270337

[22] Ju WT , Liu Y , Wang LZ , et al. Phase III trial of docetaxel cisplatin 5‐fluorouracil induction chemotherapy for resectable oral cancer suggests favorable pathological response as a surrogate endpoint for good therapeutic outcome. Cancer Commun. 2021;41:279‐283. doi:10.1002/cac2.12136 PMC7968883 33471949

[23] Bhattasali O , Han J , Thompson LDR , Buchschacher GL Jr , Abdalla IA , Iganej S . Induction chemotherapy followed by concurrent chemoradiation versus concurrent chemoradiation alone in the definitive management of p16‐positive oropharyngeal squamous cell carcinoma with low‐neck or N3 disease. Oral Oncol. 2018;78:151‐155. doi:10.1016/j.oraloncology.2018.01.031 29496043

[24] Ang KK , Zhang Q , Rosenthal DI , et al. Randomized phase III trial of concurrent accelerated radiation plus cisplatin with or without cetuximab for stage III to IV head and neck carcinoma: RTOG 0522. J Clin Oncol. 2014;32:2940‐2950. doi:10.1200/jco.2013.53.5633 25154822 PMC4162493

[25] De Felice F , Abate G , Galdieri A , Bulzonetti N , Musio D , Tombolini V . Impact of induction chemotherapy in locally advanced HPV‐negative oropharyngeal cancer. A propensity score‐matched analysis. Anticancer Res. 2016;36(6667–72):6667‐6672. doi:10.21873/anticanres.11276 27920000

[26] Vermorken JB , Remenar E , van Herpen C , et al. Cisplatin, fluorouracil, and docetaxel in unresectable head and neck cancer. N Engl J Med. 2007;357:1695‐1704. doi:10.1056/NEJMoa071028 17960012

[27] Posner MR , Hershock DM , Blajman CR , et al. Cisplatin and fluorouracil alone or with docetaxel in head and neck cancer. N Engl J Med. 2007;357:1705‐1715. doi:10.1056/NEJMoa070956 17960013

[28] Herman LC , Chen L , Garnett A , et al. Comparison of carboplatin‐paclitaxel to docetaxel‐cisplatin‐5‐flurouracil induction chemotherapy followed by concurrent chemoradiation for locally advanced head and neck cancer. Oral Oncol. 2014;50:52‐58. doi:10.1016/j.oraloncology.2013.08.007 24055193

[29] Lee KW , Koh Y , Kim SB , et al. A randomized, multicenter, phase II study of cetuximab with docetaxel and cisplatin as induction chemotherapy in unresectable, locally advanced head and neck cancer. Oncologist. 2015;20:1119‐1120. doi:10.1634/theoncologist.2015-0208 26304911 PMC4591951

[30] Lakshmaiah KC , Rudresha AH , Suresh TM , Lokanatha D , Babu GK , Jacob LA . A prospective study to assess the efficacy and toxicity of 5‐flurouracil and cisplatin versus taxane and cisplatin as induction chemotherapy in locally advanced head and neck squamous cell cancer in a regional cancer center in India. Indian J Cancer. 2015;52:65‐68. doi:10.4103/0019-509x.175601 26837977

[31] Prigge ES , Arbyn M , von Knebel DM , Reuschenbach M . Diagnostic accuracy of p16(INK4a) immunohistochemistry in oropharyngeal squamous cell carcinomas: a systematic review and meta‐analysis. Int J Cancer. 2017;140:1186‐1198. doi:10.1002/ijc.30516 27859245

[32] Grønhøj Larsen C , Gyldenløve M , Jensen DH , et al. Correlation between human papillomavirus and p16 overexpression in oropharyngeal tumours: a systematic review. Br J Cancer. 2014;110:1587‐1594. doi:10.1038/bjc.2014.42 24518594 PMC3960616

