# Supplementary figures and images for: Response to induction chemotherapy predicts survival outcomes in oropharyngeal cancer
Source: Cancer Med. 2023 Jan 27;12(8):9175–85. doi: 10.1002/cam4.5656 (PMC10166893; doi:10.1002/cam4.5656)

Supplementary Figure S1

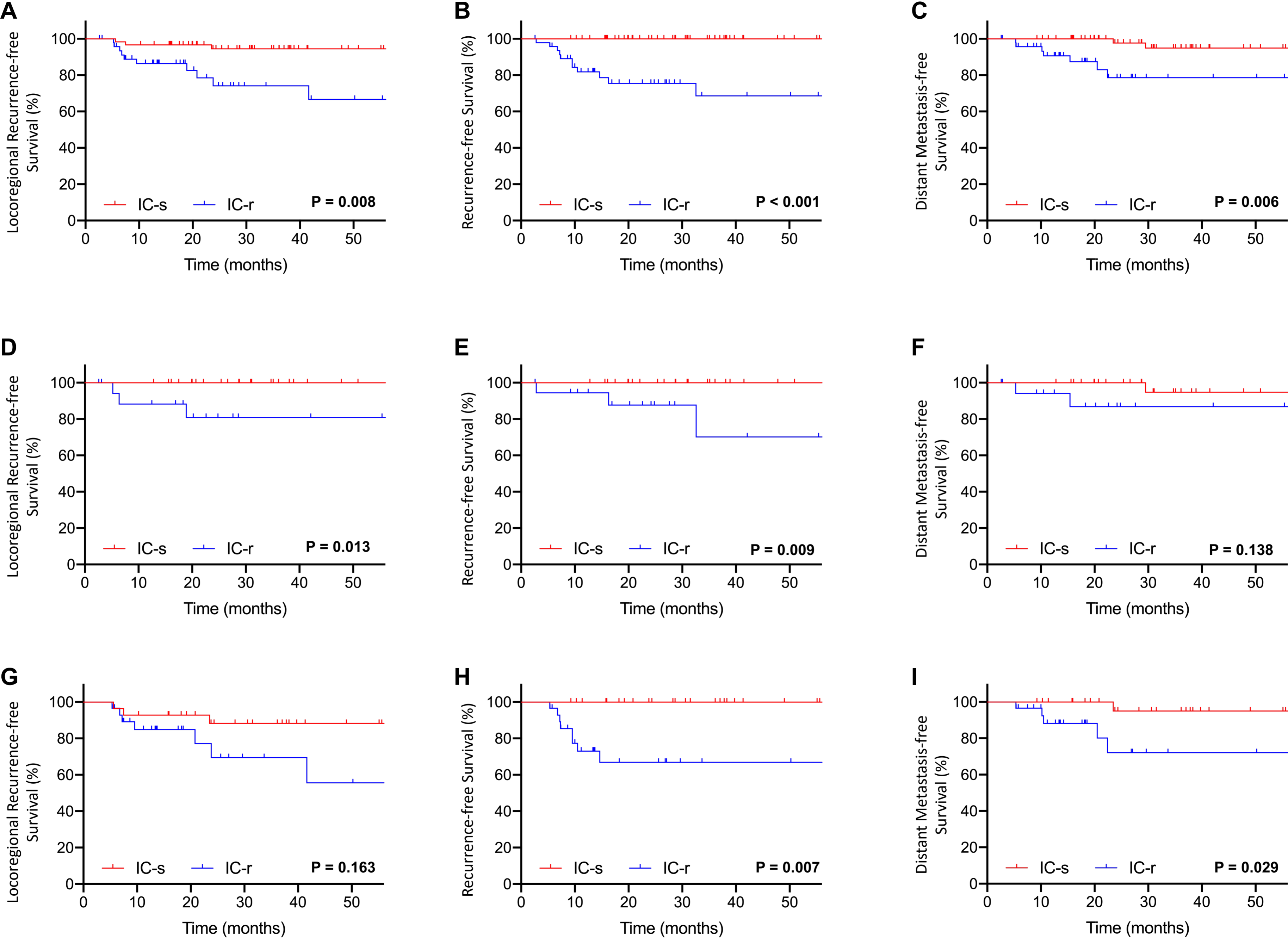

Supplement: Supplementary file 1 — Figure S1. [file CAM4-12-9175-s001.pdf]
